# Gut Microbial Profile in Asymptomatic Gallstones

**DOI:** 10.3389/fmicb.2022.882265

**Published:** 2022-06-13

**Authors:** Sen-Tao Song, Ling-Yan Cai, Xin Zeng, Wei-Fen Xie

**Affiliations:** ^1^Department of Gastroenterology, Shanghai East Hospital, Tongji University School of Medicine, Shanghai, China; ^2^Department of Gastroenterology, Shanghai Changzheng Hospital, Second Military Medical University, Shanghai, China

**Keywords:** gut microbial, asymptomatic gallstone, metabolic diseases, 16SrDNA, hypertension, non-alcoholic fatty liver disease, obesity

## Abstract

There are few studies on the changes of gut microbiota in patients with gallstones, especially in patients with asymptomatic gallstones, and there are some deficiencies in these studies, for instance, the effects of metabolic factors on gut microbiota are not considered. Here, we selected 30 asymptomatic gallstone patients from the survey population, and 30 controls according to the age and BMI index matching principle. The 16SrDNA technology was used to detect and compare the structural differences in the gut microbiota between the two groups. Compared with healthy controls, the abundance of gut microbiota in patients with gallstones increased significantly, while the microbiota diversity decreased. At the level of phylum, both groups were dominated by *Firmicutes*, *Bacteroides*, *Proteobacteria*, and *Actinobacteria*. At the genus level, there were 15 species with significant differences in abundance between the two groups. Further subgroup analysis found that only unclassified *Lactobacillales* showed differences in the intestines of gallstones patients with hypertension, non-alcoholic fatty liver disease, or patients with elevated BMI (≧24). The structure of gut microbiota in patients with gallstones changed significantly, and this might be related to the occurrence of gallstones, rather than metabolic factors such as hypertension, non-alcoholic fatty liver disease, and obesity.

## Introduction

Gallstone disease (GSD) represents a major public health problem worldwide with a prevalence ranging from 3.2 to 35.2% (Wang et al., 2018) and a frequent cause of hospital admissions, associated with high costs because of treatment and morbidity [Everhart et al., 1999; European Association for the Study of the Liver [EASL], 2016; Shabanzadeh, 2018]. Although most patients are asymptomatic during their lifetime, 10–20% will develop symptomatic gallstone disease, the consequences of which can range from simple biliary colic to potentially life-threatening complications such as cholecystitis, Gallstone ileus, choledocholithiasis, Cholestatic jaundice, cholangitis, acute biliary pancreatitis, and rarely, gallbladder cancer (Portincasa et al., 2016; Soreide, 2017). Gallstone formation is multifactorial, such as advancing age, being female, genes/ethnicity, obesity, rapid weight loss, diet, drugs, and activity. Some risk factors are unalterable, and some factors can be modified (Shaffer, 2006; Lammert et al., 2016).

The microorganisms living in the gastrointestinal cavity collectively referred to as gut microbiota or intestinal microbiota are a hotspot in recent years. The rapid advances made during the past decade suggest that the gut microbiota constitutes an important environmental factor that contributes to metabolic diseases, such as obesity, diabetes, dyslipidemia, and hypertension (Karlsson et al., 2013; Fandriks, 2017). However, those diseases are closely related to the formation of gallstones (Shaffer, 2006; Song et al., 2020), suggesting gut microbiota is likely to be associated with the gallstones. Kawai et al. (2002) found that gram-positive cocci are associated with the formation of pure cholesterol stones. It is considered that abnormal metabolism and secretion of cholesterol and bile acids as the primary pathophysiological defect in the formation of gallstones (Wang et al., 2009), and gut microbiota regulate bile acid metabolism by reducing bile acid pool size and composition (Sayin et al., 2013). Many biliary diseases are also affected by gut microbiota, such as primary biliary cholangitis/cirrhosis (Bogdanos et al., 2005; Lemoinne and Marteau, 2017) and primary sclerosing cholangitis (Tabibian et al., 2013). Meanwhile, the biliary microbiome has been proved to facilitate the formation of brown pigment stones (Wang et al., 2018). However, little is known about the correlation between gut microbiota dysbiosis and the formation of gallstones, especially which one and how the gut microbiota acts on the formation of asymptomatic gallstones. Consequently, for developing a greater understanding of the connection between GSD and the microflora communities in the body and then developing strategies a better understanding of the gut bacterial tract in asymptomatic gallstone patients is crucial.

Here, we selected 30 patients with asymptomatic gallstones from the survey population, and 30 control populations according to their age and BMI index. The 16SrDNA technology was used to detect and compare the structural differences of intestinal microbiota between two groups, to better understand the role bacteria play in the pathogenesis of asymptomatic gallstones.

## Materials and Methods

### Studied Subjects and Sample Collection

This work was based on the Three-Year Action Program of Shanghai Municipality for Strengthening the Construction of the Public Health System (no. GWIV-27.7), a cross-sectional health survey of Shanghai residents (Song et al., 2020). Among the 4,038 participants, 30 patients with asymptomatic gallstones were selected, and 30 cases without gallstones were selected according to the sex, age, and BMI index matching principle. All the subjects had no history of biliary surgery and had no history of taking antibiotics, probiotics, immunosuppressants, or traditional Chinese medicines for at least 3 months before sample collection. Criteria for diagnosis of gallstones: All the participants were examined by B-ultrasound after fasting for at least 8 h. Gallstones were identified based on the presence of strong echoes with or without acoustic shadows and rolling stone signs in the gallbladder lumen or in the gallbladder sludge. Metabolic disorders, such as hypertension, diabetes, and non-alcoholic fatty liver disease were diagnosed according to established standards (Jian-Gao and Chinese Liver Disease Association, 2010; Bakris et al., 2019; Jia et al., 2019). Body mass index (BMI) (kg/m^2^) was calculated by dividing weight (kg) by the square of height (m).

### Sample Preparation, Molecular Methods, and Bioinformatics

Stool samples were obtained and placed in a fecal collection box, which was refrigerated and transported to the laboratory by KingMed Cold Chain Logistics (Shanghai, China) within 2 h, and stored at −80°C until analysis. 16SrDNA sequencing was completed by Shanghai Majorbio Bio-pharm Technology Co., Ltd. DNA extraction was carried out according to the E.Z.N.A.^®^ DNA Kit (Omega BioTek, United States) protocol to enable amplification of the 16S V3-V4 region of the DNA gene, primers were designed based on the universal primer set, 338F: ACTCCTACGGGAGGCAGCAG; 806R: GGACTACHVGGGTWTCTAAT. The PCR products were detected and quantified with a mini fluorometer (Quantus™ Fluorometer, Promega, United States). The NEXTFLEX Rapid DNA-Seq kit (Bioo Scientific, United States) was used for library construction, and the Miseq PE300 platform of Illumina was used for sequencing. The quality control of the original sequencing sequence was performed by Trimmomatic software. Spliced by FLASH software, after filtering and removing the low-quality and short-length sequences, the UCHIME software was used to eliminate the chimera and obtain valid sequence data. The UPARSE software (version 7.1^1^) was used to perform OTU clustering and analysis of sequences based on 97% similarity. The alpha diversity analysis was conducted to reveal the microbial diversity indices, such as the Chao, ACE, and Simpson diversity indices. The Wilcoxon signed-rank test was used to test the significance among groups on these indices. The beta diversity analysis was carried out using UniFrac to compare the results of PCoA at the OTU level with the community ecology package, R-forge (vegan 2.0 package was used to generate a PCA figure; the differences among the groups were tested). The hierarchical clustering analysis was performed using the Primer 5 software (Primer-E Ltd., United Kingdom). The original data are accessible at NCBI-BioProject under the accession number PRJNA822035.

### Statistical Methods

Statistical analyses were performed by using SPSS version 18 (IBM, Armonk, NY, United States). Measurement data were presented as mean ± standard deviation (SD). Comparison between the two groups was performed by the Wilcoxon rank-sum test. Statistical significance was set at *p ≤ 0.05, ^**^p ≤ 0.01, and ^***^p ≤ 0.001. *p* ≤ 0.05 was considered statistically significant.

## Results

### Demographic Characteristics

A total of 60 subjects (30 in the asymptomatic gallstone group and 30 in the control group) were analyzed. As shown in Table 1, the two groups were comparable with respect to the clinical characteristics of age, sex, BMI, hypertension, diabetes, NAFLD, and Mets. There were also no significant differences in the laboratory results, such as lipid metabolism index, glucose metabolism index, and liver function.

**TABLE 1:** Clinical characteristics of the subjects.

Characteristics	Asymptomatic gallstone group *n* = 30	Control group *n* = 30	*p*-value
Sex			
Male, No.	12	12	1.000
Female, No.	18	18	
Age (y)	52.58 (7.94)	53.02 (8.25)	0.833
Hypertension	17	13	0.731
T2DM	3	2	1.000
NAFLD	12	8	0.412
MetS	7	7	1.000
BMI (kg/m^2^)	24.66 (3.70)	24.46 (3.50)	0.835
WHtR	0.51 (0.06)	0.51 (0.04)	0.896
Waist (cm)	84.21 (10.10)	84.16 (7.31)	0.985
TC (mg/dL)	5.16 (1.03)	5.78 (1.31)	0.052
LDL (mg/dL)	3.19 (0.91)	3.68 (1.20)	0.094
HDL (mg/dL)	1.49 (0.45)	1.49 (0.41)	0.994
Triglyceride (mg/dL)	1.63 (1.12)	2.03 (2.22)	0.405
AST (IU/L)	21.45 (7.17)	21.40 (5.39)	0.973
ALT (IU/L)	21.31 (10.75)	24.79 (15.69)	0.352
GGT (IU/L)	25.90 (14.72)	44.92 (82.55)	0.248
ALP (IU/L)	80.72 (28.06)	88.44 (60.37)	0.537
FPG (mmol/L)	5.51 (1.03)	5.97 (2.04)	0.281
2Hpg (mmol/L)	6.81 (2.92)	5.84 (1.65)	0.146
HbA1c (%)	5.60 (0.45)	5.58 (1.04)	0.930

*NAFLD, Non-alcoholic fatty liver disease; BMI, body mass index; WHtR, waist-height ratio; TC, total cholesterol; HDL, high-density lipoprotein-cholesterol; LDL, low-density lipoprotein-cholesterol; AST, aspartate transaminase; ALT, alanine transaminase; GGT, gamma-glutamyltransferase; ALP, alkaline phosphatase; FPG, fasting plasma glucose; 2hPG, 2-h postprandial blood glucose; HbA1c, glycosylated hemoglobin.*

### Gut Microbial Dysbiosis in Asymptomatic Gallstone Patients

**FIGURE 1:**
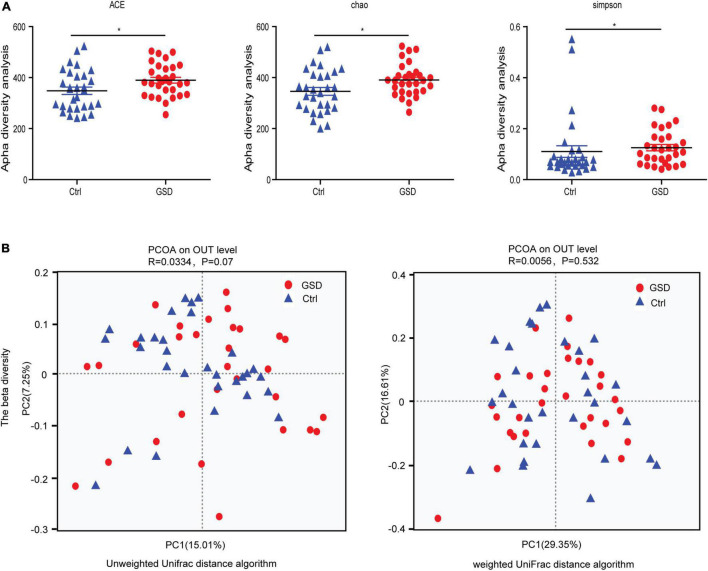
Microbial community characteristics generated by 16SrDNA sequencing. **(A)** Boxplots of the alpha-diversity indices in the asymptomatic gallstone patients and controls. From left to right, observed ACE, Chao, and Simpson index. **(B)** Principal coordinates analysis showed the composition of the asymptomatic gallstone patients and controls. *P < 0.05.

Since the development of microbiota-targeted therapies is an intriguing new avenue for many diseases, changes in microbiome composition or function must first be carefully shown to contribute to GSD. To investigate the specific changes of microbiota in samples from asymptomatic gallstone patients, we performed 16SrDNA sequencing on samples collected from all the participants. We obtained a total of 3,579,322 effective sequences and 1,479,043,036 base pairs from DNA extracted from fecal samples of asymptomatic gallstone patients (*n* = 30) and controls (*n* = 30). The average length of each sequence was 413.2 base pairs. To investigate the diversity of gut microbiota in the two groups, we used the alpha diversity analysis. The results showed that the abundance of gut microbiota in the asymptomatic gallstones group was significantly increased compared with the control group (median chao1 index of 390.57 vs. 346.07 in the controls, *p* = 0.0364; median ace index of 389.65 vs. 348.32 in the controls, *p* = 0.0292), while the diversity of gut microbiota was decreased (median Simpson index of 0.13 vs. 0.11 in the controls, *p* = 0.0183) (Figure 1A). The principal coordinates analysis showed that there was no significant difference in beta diversity between the two groups (Figure 1B).

**FIGURE 2:**
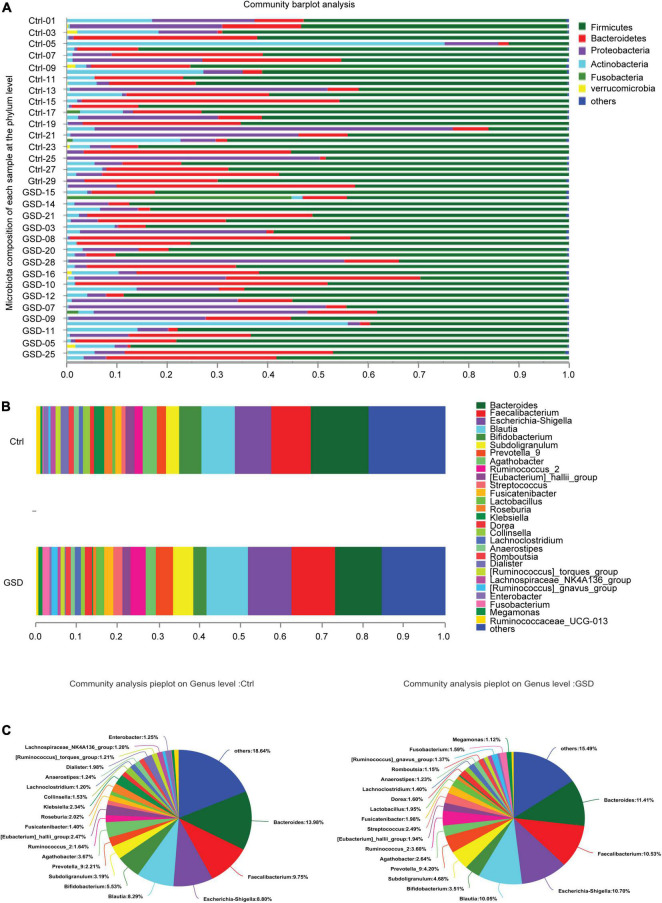
The microbiota composition of each sample at the genus and phylum level. **(A)** Microbiota composition of each sample at the phylum level. **(B,C)** The proportion of gut microbiota abundance in two groups (genus level). GSD, asymptomatic gallstone group; Ctrl, control group. *P < 0.05; **P < 0.01.

We next investigated the richness and evenness of the gut microbiota in all samples at the genus, species, and gene levels. At the phylum level, the top 4 abundance in descending order were *Firmicutes*, *Bacteroidetes*, *Proteobacteria*, and *Actinobacteria*. The average abundance of each was 62.63, 17.60, 12.51, and 5.37% in the gallstone group and 59.62, 18.64, 13.78, and 7.52% in the control group (Supplementary Table 1). However, there were also some samples with large variations in the abundance of the microbiota. For example, the abundance of *Actinobacteria* increased significantly in control-05, and *Proteobacteria* was the main bacterial phylum in the control-20 (Figure 2A).

According to the proportion of microbiota abundance, the main groups ranked forward were *Bacteroides*, *Faecalibacterium*, *Escherichia-Shigella*, *Blautia*, *Bifidobacterium*, *Prevotella_9*, *Subdoligranulum*, *Agathobacte*, *Ruminococcus_2*, and *[Eubacterium]_hallii*_group at the genus level, while there was little difference between in 2 groups (Figure 2B). However, there was an increment abundance of *Streptococcus*, *Lactobacillus*, *Dorea*, *Romboutsia*, *Fusobacterium*, and *Megamonas*, while decrement of *Klebsiella*, *Roseburia*, *Collinsella*, *Dialister*, and *Enterobacer*in asymptomatic gallstone patients (Figure 2C).

**FIGURE 3:**
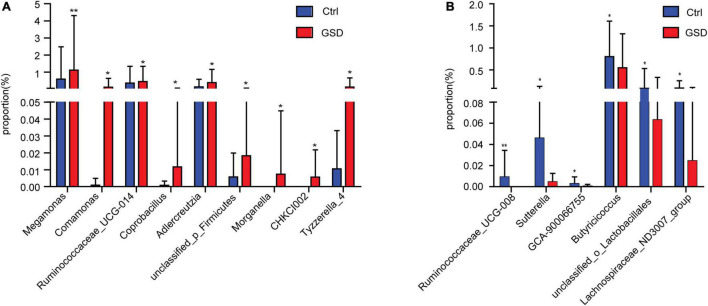
Comparison of gut microbiota abundance between asymptomatic gallstone group and control group at the genus level. **(A)** Genera with increasing abundance in asymptomatic gallstone group. **(B)** Genera with decreasing abundance in asymptomatic gallstone group. *P < 0.05; **P < 0.01.

Further abundance analysis showed that there were 15 genera with significant differences between the two groups at the genus level. *Megamonas*, *Comamonas*, *Ruminococcaceae_UCG*-014, *Coprobacillus*, *Adlercreutzia*, unclassified_*p_Firmicutes*, *Morganella*, *CHKCI002*, and *Tyzzerella_4* were in relatively higher abundance in asymptomatic gallstone groups. *Ruminococcaceae_UCG-008*, *Sutterella*, *GCA-900066755*, *Butyricicoccus*, unclassified_*o_Lactobacillales*, and *Lachnospiraceae_ND3007_*group were significantly lower abundance in asymptomatic gallstone groups. Moreover, there were no *Morganella* and *CHKCI002* detected in control patients and no *Ruminococcaceae_UCG-008* in asymptomatic gallstone patients. It suggested that the declined abundance of *Ruminococcaceae_UCG-008* and increased abundance of *Morganella* and *CHKCI002* may act on the formation of GSD (Figures 3A,B).

### Gut Microbial Dysbiosis in Asymptomatic Gallstone Patients Accompanied by Other Diseases

**FIGURE 4:**
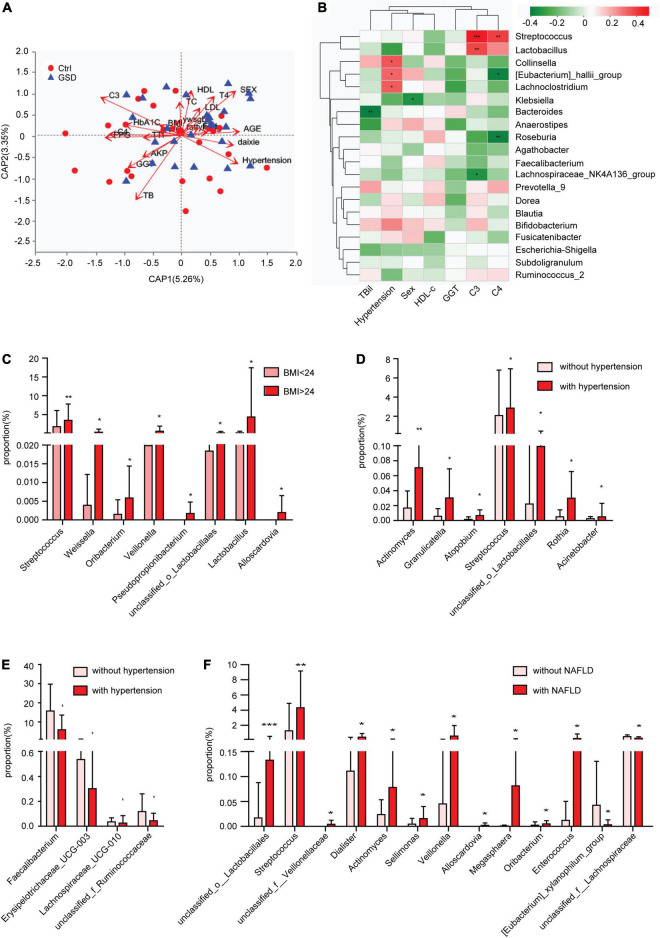
Gut microbial dysbiosis in asymptomatic gallstone patients accompanied with other diseases. **(A)** Influence index with distance-based redundancy analysis. **(B)** The relationship between different species (the top 20 abundance, *p* < 0.1 and VIF < 10) and clinical factors. **(C)** Differences of gut microbiota composition in asymptomatic gallstone patients with BMI < 24 or BMI ≧ 24 (genus level). **(D,E)** Differences of gut microbiota composition in asymptomatic gallstone patients with or without hypertension (genus level). **(F)** Differences of gut microbiota composition in asymptomatic gallstone patients with or without NAFLD (genus level). *P < 0.05; **P < 0.01.

As we all know, hypertension and obesity are important risk factors for gallstones. Recently, the immune system was considered to be involved in cholesterol cholelithogenesis (Maurer et al., 2009). Here, we found that gender, hypertension, TBil, C3, C4, FPG, and GGT had a great influence on the results of flora with distance-based redundancy analysis (Figure 4A, Table 2 and Supplementary Table 2). The correlation analysis showed that *Streptococcus* and *Lactobacillus* were positively correlated with complement C3 and C4, while *Roseburia* and *Eubacterium _ hallii_* Group were negatively correlated with C4 level. In addition, *Eubecterium_hallii_*Group and *Lachnoclostridium*, *Collinella* were positively correlated with hypertension (Figure 4B). To determine the effect of other diseases on microbial composition, we further analyzed the subgroups of patients with elevated BMI (≧24), non-alcoholic fatty liver disease, and hypertension in the gallstone group. At the genus level, there were eight bacterial taxa that displayed different abundance between asymptomatic gallstone patients with BMI ≧ 24 and BMI < 24, and all of them were increased in asymptomatic gallstone patients accompanied by BMI ≧ 24 (Figure 4C). Moreover, 11 bacterial taxa were found to display different abundance between asymptomatic gallstone patients accompanied by hypertension and without hypertension, while 7 were increased (Figure 4D) and 4 were decreased in asymptomatic gallstone patients accompanied by hypertension (Figure 4E). There were 13 bacterial taxa that displayed different abundance between asymptomatic gallstone patients accompanied by NAFLD and without NAFLD. Among them, 11 genera were enriched and 2 genera were decreased in asymptomatic gallstone patients accompanied by NAFLD. The abundance of Streptococcus in asymptomatic gallstone patients accompanied by NAFLD was mostly significantly ascended (Figure 4F). Furthermore, we found that among the 15 species with significant differences in genus level between the two groups, none of them showed differences in the three subgroups except unclassified*_ Lactobacillales*.

**TABLE 2:** Correlation coefficient between clinical factors and gut microbial.

	VIF values	*r*^2^ values	*p* values
TB	1.84	0.22	0.003
C3	3.42	0.21	0.003
SEX	3.70	0.14	0.027
FPG	> 10	0.12	0.047
Hypertension	1.35	0.11	0.048
C4	2.17	0.11	0.06
GGT	7.42	0.10	0.077
HDL	3.03	0.09	0.101
T4	8.83	0.08	0.135
HbA1C	2.61	0.07	0.181
AGE	2.36	0.07	0.184
ALP	7.57	0.05	0.317
TC	3.23	0.05	0.323
LDL	1.89	0.04	0.405
BMI	5.42	0.01	0.722
T3	5.41	0.01	0.733
Waist	6.27	0.01	0.795
FT4	5.77	0.01	0.852
NAFLD	2.45	0.001	0.965

*TB, total bilirubin; T3, Triiodothyronine; FT4, free thyroxine.*

## Discussion

It is now well accepted that the pathogenesis of GSD is determined by genetic background, hepatic hypersecretion of biliary cholesterol, rapid precipitation of solid cholesterol crystals in bile, gallbladder dysmotility, and intestinal factors (with increased absorption of cholesterol, slow intestinal motility, and dysbiosis) (Di Ciaula et al., 2018). Intestinal dysbiosis makes a significant contribution to the development of GSD. Many reports are underlining the association of the gut microbiota with the pathogenesis of GSD in mice and humans. It was found that in the feces of mice with cholesterol gallstones (induced by a lithogenic diet), the *Firmicutes/Bacteroidetes* ratio and the *Firmicutes* content decreased, and the richness and alpha diversity of the microbiota also significantly reduced (Wang et al., 2017). Studies with experimental animal models indicated that *enterohepatic helicobacters* promote the formation of cholesterol gallstones *in vivo* (Maurer et al., 2005). The cholecystectomy could affect the gut microbiota, and the abundance of *B. obeum* and *V. parvula*, which have azoreductase activity, was increased in the cholecystectomy group (Yoon et al., 2019). Wang et al. (2020) found that the diversity of intestinal bacteria and the abundances of certain phylogroups significantly decreased, especially *Firmicutes* in the gallstone group. Keren et al. (2015) showed that intestinal microbial diversity, the abundances of the genus *Roseburia* and the species Bacteroides uniformis were decreased, and those of the family *Ruminococcaceae* and the genus *Oscillospira* were increased in patients with gallstones compared with the controls. Gallstone patients had higher overall concentrations of fecal bile acids, and *Oscillospira* may predispose individuals to cholesterol gallstones for it correlated negatively with primary bile acids and fecal cholesterol concentration and positively with the secondary bile lithocholic acid in the feces. In our study, we focused on the asymptomatic gallstone patients and found that the abundance of gut microbiota in the asymptomatic gallstones group was significantly increased compared with the control group, while the diversity of gut microbiota was also decreased. Although the main bacteria composition of the two groups is similar, there was an increment abundance of *Streptococcus*, *Lactobacillus*, *Dorea*, *Romboutsia*, *Fusobacterium*, and *Megamonas*, while decrement of *Klebsiella*, *Roseburia*, *Collinsella*, *Dialister*, and *Enterobacer*in asymptomatic gallstone patients. Some of them were consistent with many studies (Grigor’eva, 2020). Previous studies have shown that obesity is associated with an elevated *Firmicutes/Bacteroidetes* ratio in the gut microbiota (Grigor’eva, 2020), and obesity is a major risk factor for developing GSD. However, there was no significant difference in *Firmicutes/Bacteroidetes* ratio between the asymptomatic gallstone patients (3.56) and controls (3.20) in our study.

The human gut microbiota was composed of nine kinds of bacterial phyla, and dominated by four: *Bacteroidetes*, *Firmicutes*, *Actinobacteria*, and *Proteobacteriat* (Tap et al., 2009). Although there was no significant difference of the four bacterial phyla between the asymptomatic gallstone patients and controls in our study, we found that the abundance of *Ruminococcaceae_UCG-008*, *Sutterella*, *GCA-900066755*, *Butyricicoccus*, unclassified*_o_Lactobacillales*, and *Lachnospiraceae_ND3007_*group were significantly decreased in asymptomatic gallstone patients at the genus level. Among them, *Ruminococcaceae_UCG-008*, *Butyricicoccus*, *Butyricicoccus*, unclassified*_o_Lactobacillales*, and *Lachnospiraceae_ND3007_*group belong to the phylum of *Firmicutes*. *Butyricicoccus* species are considered autochthonous microbes predominantly colonizing the mucosa-associated mucus layer of the colon of mice and humans. It was decreased in patients with inflammatory bowel disease (Eeckhaut et al., 2013). The evidence from different studies shows that *Lachnospiraceae* might influencemetabolic syndrome, obesity, diabetes, liver diseases, IBD, and CKD (Vacca et al., 2020). *Megamonas* has not previously been reported as a dominant genus in gut microbiome studies with European and American subjects but was found in studies with Chinese individuals. The genus *Megamonas* was detected as highly abundant in colorectal cancer (Yachida et al., 2019). *Morganella* is an opportunistic secondary invader, acting on summer diarrhea, was founded in many cases (such as brain abscess, septicemia, tubo-ovarian abscess, and postoperative foot infection in a diabetic). It has been isolated from human urine, gallbladder, stool, sputum, and other respiratory samples, and assorted wound sites (O’Hara et al., 2000). The *Comamonas* have been shown to be capable of catabolizing a wide range of organic substrates, such as amino acids, carboxylic acids, steroids, and aromatic compounds. Some *Comamonas* species have been suggested to be involved in some invasive infections, like appendicitis, bacteremia, and meningitis (Wu et al., 2018). All *Megamonas*, *Morganella*, and *Comamonas* were significantly increased in asymptomatic gallstone patients. This indicated that the occurrence of gallstones may be related to the increase of pathogenic bacteria and the decrease of beneficial bacteria.

Our previous study revealed that metabolic diseases (such as hypertension and obesity) were closely related to the formation of gallstones (Song et al., 2020). Studies have shown that obesity and NAFLD were associated with an elevated *Firmicutes/Bacteroidetes* ratio in the gut microbiota (Leung et al., 2016; Grigor’eva, 2020). Evidence from the fecal microbiota transplantation (FMT) and interventions that target the gut microbiota, such as a high-fiber diet, probiotics, and antibiotics, reflected that gut dysbiosis plays a role in the genesis of hypertension (Li et al., 2017; Wilck et al., 2017; Yang et al., 2018). On the other hand, animal studies in which the gut microbiota are manipulated, and observational studies in patients with NAFLD, have provided considerable evidence that dysbiosis contributes to the pathogenesis of NAFLD. All of this indicated that the change in gut dysbiosis in our study may be related to NAFLD, obesity, or hypertension. Distance-based redundancy analysis also showed that hypertension, C3, and C4 had a great influence on the results of flora. Here, streptococcus and lactobacillus were positively correlated with complement C3 and C4, while *Roseburia* and *Eubacterium_hallii_*Group were negatively correlated with the C4 level. In addition, *Eubecterium_hallii_*Group and *Lachnoclostridium*, *Collinella* were positively correlated with hypertension. The abundance of *Klebsiella* in men is higher than that in women. However, among the 15 species with significant differences in genus level in the asymptomatic gallstone patients, there were only unclassified-*o-Lactobacillales* significantly different between the patients with BMI≧24 or BMI < 24, with or without hypertension, with or without NAFLD. These indicate that most of the 15 species are specially related to gallstones. *Streptococcus* significantly increased in the asymptomatic gallstone patients with BMI≧24, hypertension, or NAFLD. It revealed that *Streptococcus* plays an important role in the pathogenesis of obesity, hypertension, and NAFLD.

In conclusion, our data confirmed that the diversity of the gut microbiota in asymptomatic gallstone patients was decreased, while the abundance was significantly increased compared with the control group. The gut microbiota in patients with asymptomatic gallstone have shown a significant increase in the phyla Firmicutes (*Megamonas*, *Coprobacillus*, *Ruminococcaceae_UCG-014*, unclassified*_p_Firmicutes*, *Tyzzerella_4*), Actinobacteria (*Adlercreutzia*, *CHKCI002*), Proteobacteria (*Comamonas*, *Morganella*), and a significant decrease in the phyla Firmicutes (*Ruminococcaceae_UCG-008*, unclassified*_o_Lactobacillales*, *GCA-900066755*, *Butyricicoccus*, *Lachnospiraceae_ND3007_*group), and Proteobacteria (*Sutterella*). Most of them are especially associated with the asymptomatic gallstone, not with obesity, NAFLD, and hypertension.

## Data Availability Statement

The datasets presented in this study can be found in online repositories. The names of the repository/repositories and accession number(s) can be found below: https://www.ncbi.nlm.nih.gov/bioproject/, PRJNA822035.

## Ethics Statement

The studies involving human participants were reviewed and approved by the Ethics Committee of Changzheng Hospital (no. 2016SL039). The patients/participants provided their written informed consent to participate in this study.

## Author Contributions

XZ and L-YC obtained funding. S-TS and L-YC designed the study and wrote the manuscript. S-TS performed the experiments and contributed to the analysis and interpretation of data. L-YC performed the statistical analysis. XZ provided material support. W-FX proposed initial proposal and revised the manuscript.

## Conflict of Interest

The authors declare that the research was conducted in the absence of any commercial or financial relationships that could be construed as a potential conflict of interest.

## Publisher’s Note

All claims expressed in this article are solely those of the authors and do not necessarily represent those of their affiliated organizations, or those of the publisher, the editors and the reviewers. Any product that may be evaluated in this article, or claim that may be made by its manufacturer, is not guaranteed or endorsed by the publisher.

## Footnotes

1
http://drive5.com/uparse/
